# A case of celiac plexus block causing iatrogenic Cushing's syndrome

**DOI:** 10.1002/ccr3.8777

**Published:** 2024-04-15

**Authors:** Ana‐Maria Chindris, Sarika N. Rao, Razvan M. Chirila, Adrian G. Dumitrascu

**Affiliations:** ^1^ Division of Endocrinology, Department of Internal Medicine Mayo Clinic Jacksonville Florida USA; ^2^ Department of Internal Medicine Mayo Clinic Jacksonville Florida USA

**Keywords:** adrenal insufficiency, corticosteoids, Cushing's, exogenous, iatrogenic

## Abstract

Treatment with corticosteroids can lead to iatrogenic Cushing's syndrome when used for longer intervals and in high doses. Less common administration routes may conceal the exposure, raising the possibility of misdiagnosis and mismanagement.

## 1 INTRODUCTION

Corticosteroids, widely prescribed for their anti‐inflammatory and immunosuppressive properties, can have significant side effects when used in supraphysiological doses.^1^ One of the key concerns is the suppression of the hypothalamic‐pituitary‐adrenal (HPA) axis,^2^ along with symptoms of hypercortisolism (iatrogenic Cushing's syndrome).^3^, ^4^

Differentiating between exogenous and endogenous Cushing's syndrome is typically straightforward and involves a thorough review of the patient's medical history and medications, which often reveals corticosteroid usage. Adverse effects associated with excess of glucocorticoid activity are countless and widespread. In iatrogenic Cushing's, they are more likely to occur with oral and parenteral routes of administration. Topical administration of steroids is often used in the attempt to eliminate or at least minimize the systemic effects; nevertheless, cases of overt iatrogenic Cushing's syndrome have been reported with virtually all types of steroid formulations.

Injectable medication cocktails, comprising a combination of long‐acting corticosteroids and local anesthetics, have been increasingly used in the management of chronic pain, offering an alternative to opioids.^5^, ^6^ Despite their frequent use, many patients remain unfamiliar with the medications they receive, and with their potential side effects. In addition, physicians often focus on questioning the patient regarding more common procedures, such as perivertebral, intra‐, or periarticular injections, but may not be aware of less frequent ones such as celiac plexus blocks (CPB) for chronic abdominal pain. This gap in knowledge and communication can lead to misinterpretation of symptoms and test results, potentially resulting in mismanagement of the patient's condition.

## 2 CASE HISTORY

A 62‐year‐old female with a past medical history significant for dysphagia, gastroesophageal reflux disease, recurrent pancreatitis, hyperlipidemia, hypertension, and migraines was referred to the endocrinology clinic for recent onset fatigue, weakness, and decreased appetite as well as a history of unintentional weight gain and mylagias. She had just completed a cardiology visit for fluctuating blood pressure and untreated hyperlipidemia.

Her medications include omeprazole 20 mg daily, pancrelipase 24,000 unit, three capsules three times daily; rosuvastatin 20 mg daily, amlodipine 10 mg daily, atenolol 25 mg daily, and candesartan 8 mg twice daily. The rosuvastatin and candesartan were prescribed at the recent cardiology visit.

On examination, she was hypertensive, blood pressure 172/82 mmHg with a pulse of 72 bpm. She was obese, with a BMI of 34.1 kg/m^2^; and according to the chart review, her weight had increased from 94 to 99.5 kg over 4 months. She exhibited facial plethora, central obesity, dorsocervical, and supraclavicular fat pad. No purple stria or evidence of hirsutism was present. She reported easy bruising, but no significant ecchymosis was noted at the time of the skin exam. The remainder of the physical examination was unremarkable.

## 3 METHODS

Laboratory testing included the following (reference ranges listed in parentheses): TSH 3.3 mIU/L (0.3–4.2 mIU/L), serum morning cortisol <1.0 μg/dL (7–25 μg/dL), adrenocorticotropic hormone (ACTH) <5.0 pg/mL (7.2–63 pg/mL), prolactin 15.6 ng/mL (4.8–23.3 ng/mL), and hemoglobin A1C 5.4% (4.0–5.6) with a fasting morning glucose of 102 mg/dL (70–100 mg/dL). The lipid profile demonstrated an elevated total cholesterol of 252 mg/dL (<200 mg/dL), LDL of 180 mg/dL (<100 mg/dL), HDL of 56 mg/dL (>60 mg/dL) and triglycerides of 81 mg/dL (<150 mg/dL).

A thorough review of her medication history showed no evidence of prescribed glucocorticoids, despite laboratory test results strongly indicating their presence. The patient denied any use of nutritional supplements, over‐the‐counter steroid‐containing topical preparations, or corticosteroids from other medical providers. Eventually, further discussions with her medical teams uncovered that she had been undergoing CBP procedures, every few months, for the past 2 years. The injectable medication cocktail intended to manage abdominal pain related to chronic pancreatitis, contained 80 mg triamcinolone acetonide and 20 mL bupivacaine. The patient was not aware that these injections contained corticosteroids and of the potential associated side effects. Notably, her ACTH and cortisol levels were measured 1 week following the most recent CPB.

## 4 FOLLOW‐UP

Three months later, repeat testing demonstrated normal AM cortisol levels of 13 μg/dL and ACTH levels of 17 pg/mL, along with a normal Cortrosyn stimulation test consistent with full recovery of the HPA axis. The patient was counseled regarding risk of side effects related to prolonged exposure to high‐dose corticosteroids and recommended to discuss with her providers decreasing the dose and frequency of treatments to minimize these risks.

## 5 DISCUSSION

Corticosteroids have numerous applications in treating inflammation and autoimmune conditions.^1^ When used in pharmacologic doses, these agents can cause numerous adverse effects associated with an excess of glucocorticoid activity.^3^ Transient or sometimes permanent adrenal insufficiency can also occur, as a result of suppression of the HPA axis. This may result in a mixed clinical presentation that includes both stigmata of Cushing's syndrome and symptoms of adrenal insufficiency.

Early distinction between endogenous and exogenous (iatrogenic) Cushing's is essential, as the workup and management are completely different. In endogenous Cushing's, the assessment entails confirming the state of hypercortisolism followed by determining ACTH dependence and then identifying and treating the cause such as removal of an ACTH producing pituitary adenoma or of an autonomously functioning adrenal adenoma. This workup is often tedious, expensive, and time‐consuming. On the contrary, in patients with exogenous Cushing's, the aim is to explore nonsteroid management options for their underlying condition or, if this is not an option, to use the lowest steroid dose that provides symptom control.

A detailed history and medication review will typically reveal the corticosteroid therapy and no further laboratory testing is necessary, but if obtained, the ACTH and serum cortisol will be undetectable in most cases, consistent with HPA axis suppression, such as in the case of our patient. However, in situations where the timing of the laboratory testing coincides with partial recovery of the HPA axis, lack of awareness of the steroid exposure may trigger additional unnecessary workup. While exceedingly rare, both entities can coexist.^7^

Secondary adrenal insufficiency from prolonged HPA axis suppression can also occur and is often overlooked in clinical practice. While our patient's HPA axis recovered completely by 3 months, according to a recent meta‐analysis of 74 studies, the incidence of secondary adrenal insufficiency ranged from 4.2% with nasal corticosteroids to 52.2% for intra‐articular steroids.^2^ The risk of iatrogenic Cushing's is dependent of dose, duration, specific agent, and individual susceptibility. A large population‐based study set to investigate dose‐related risks of adrenal insufficiency, iatrogenic hypercortisolism, and mortality in adults receiving oral corticosteroids for chronic inflammatory illnesses, found that for every increase in daily dose of 5 mg prednisone (or equivalent), the risk increased by 7% for adrenal insufficiency, 9% for Cushing's syndrome, and 6% for mortality.^3^ Oral and parenteral steroids are more commonly associated with symptoms of hypercortisolism; nevertheless, this can occur with virtually all pharmacological formulations (Table 1).^8^, ^9^, ^10^, ^11^, ^12^, ^13^

**TABLE 1:** Summary of representative case reports and case series of iatrogenic Cushing's related to topical administration of steroids and over the counter supplements.

Year published	Article type	Medication used	Route of administration	Patient age	Citation
2010	Case report	Clobetasol propionate	Epicutaneous	8 months	[8]
2014	Case series	Clobetasol propionate	Epicutaneous	51–62 years	[9]
2015	Case report	Dexamethasone (0.1%)	Eye drops	1.5 months	[10]
2018	Case series	Dexamethasone	Intranasal	1.5–3 months	[11]
2023	Case report	Over the counter supplement containing dexamethasone	Oral	35 years	[12]
2023	Case report	Triamcinolone acetonide	Celiac plexus block	27 years	[13]

Celiac plexus block has been increasingly used for the past decade for treatment of abdominal pain related to pancreatic cancer and chronic pancreatitis. The injection cocktail typically consists of bupivacaine 20 mL and 40–80 mg of triamcinolone (at the endoscopist's discretion), and the patients typically require repeated treatments, every 3–4 months.^14^ To date, there are no published studies assessing the incidence of Cushing's syndrome and adrenal insufficiency associated with CPB, but a recent case report of Cushing's syndrome after CPB was recently reported.^13^

Physician awareness of the steroid exposure is critical for establishing the correct diagnosis and for appropriately managing the comorbidities related to hypercortisolism (i.e., diabetes, hypertension, obesity, osteoporosis). Sometimes, this requires additional record review and reaching out directly to the patient's other treating providers. A second critical step in the management includes seeking alternative, steroid‐sparing treatment options, or if that is not feasible, using the lowest steroid dose and frequency possible.

## 6 CONCLUSION

When suspected clinically, a thorough inquiry regarding corticosteroid therapy should be conducted to confirm the correct diagnosis of iatrogenic hypercortisolism. Additionally, patients undergoing treatment with therapeutic doses of corticosteroids should be regularly and thoroughly counseled about the potential adverse effects of prolonged use.

## AUTHOR CONTRIBUTIONS

**Ana‐Maria Chindris:** Conceptualization; writing – original draft; writing – review and editing. **Sarika N. Rao:** Writing – review and editing. **Razvan M. Chirila:** Conceptualization; writing – review and editing. **Adrian G. Dumitrascu:** Writing – original draft; writing – review and editing.

## FUNDING INFORMATION

This study did not receive any specific funding support.

## CONFLICT OF INTEREST STATEMENT

The authors have no conflict of interest to declare.

## CONSENT

Written informed consent was obtained from the patient to publish this report in accordance with the journal's patient consent policy.

## Data Availability

All data underlying the results are available as part of the article, and no additional source data are required.
